# Bayesian multi-QTL mapping for growth curve parameters

**DOI:** 10.1186/1753-6561-4-s1-s12

**Published:** 2010-03-31

**Authors:** Henri C M Heuven, Luc L G Janss

**Affiliations:** 1 Clinical Sciences of Companion Animals Faculty of Veterinary Medicine, Utrecht University P.O. box 80163, 3508 TD Utrecht, The Netherlands; 2 Animal Breeding and Genomics Centre, Wageningen University P.O. box 338, 6700AH Wageningen, the Netherlands; 3 Aarhus University DJF Department of Genetics and Biotechnology P.O. Box 50, 8830 Tjele, Denmark.

## Abstract

**Background:**

Identification of QTL affecting a phenotype which is measured multiple times on the same experimental unit is not a trivial task because the repeated measures are not independent and in most cases show a trend in time. A complicating factor is that in most cases the mean increases non-linear with time as well as the variance. A two- step approach was used to analyze a simulated data set containing 1000 individuals with 5 measurements each. First the measurements were summarized in latent variables and subsequently a genome wide analysis was performed of these latent variables to identify segregating QTL using a Bayesian algorithm.

**Results:**

For each individual a logistic growth curve was fitted and three latent variables: asymptote (ASYM), inflection point (XMID) and scaling factor (SCAL) were estimated per individual. Applying an 'animal' model showed heritabilities of approximately 48% for ASYM and SCAL while the heritability for XMID was approximately 24%. The genome wide scan revealed four QTLs affecting ASYM, one QTL affecting XMID and four QTLs affecting SCAL. The size of the QTL differed. QTL with a larger effect could be more precisely located compared to QTL with small effect. The locations of the QTLs for separate parameters were very close in some cases and probably caused the genetic correlation observed between ASYM and XMID and SCAL respectively. None of the QTL appeared on chromosome five.

**Conclusions:**

Repeated observations on individuals were affected by at least nine QTLs. For most QTL a precise location could be determined. The QTL for the inflection point (XMID) was difficult to pinpoint and might actually exist of two closely linked QTL on chromosome one.

## Background

Identification of QTL affecting a phenotype which is measured multiple times on the same experimental unit is not a trivial task because the repeated measures are not independent and in most cases show a trend in time. A complicating factor is that in most cases the mean increases non-linear with time as well as the variance, e.g. growth or yield. Another example is behavior, where a questionnaire involving many items is used to describe the phenotype, e.g. aggression. Also in this case multiple measurements have to be combined in order to detect QTL affecting such a trait. Mapping the genetic architecture of such a dynamic complex trait is called functional mapping and was reviewed by Wu and Lin [1]. Yang and Xu [2] applied functional mapping using a Bayesian shrinkage analyses with Legendre polynomials, which has the advantage that it will fit any trend in time but can be harder to interpret biologically.

Although simultaneous estimation of aggregate parameters and QTL affecting them in a hierarchical model would be best it will be difficult to implement it especially if genome wide marker data has to be analyzed. Therefore a two-step approach was used: first the repeated observations were summarized in latent variables and subsequently a genome wide analysis was performed using these latent variables as phenotypes.

The objective of this study was to identify segregating QTL affecting a simulated phenotype that was repeatedly measured on each individual, using a Bayesian algorithm.

## Methods

In a 2 generation pedigree 5 males were combined with 20 females and produced 100 full sib families of 20 members each. Fifty percent of the families were repeatedly phenotyped for a yield trait. The five measurements were taken on day 1, 132, 265, 397 and 530. A full description of the dataset can be found at the website of XIII^th^ QTLmas workshop (http://www.qtlmas2009.wur.nl/UK/Dataset/).

### Latent variable analysis

A logistic growth function was fitted to five measurements obtained on each of the 1000 individuals that were phenotyped using R [3]. A curve for each individual was fitted and the parameters were stored using the following model

Y_ij_ = (asym + as_i_) / (1 + exp( (t - (xmid + xm_i_)) / (scal + sc_i_)) + e_ij_

Where Y_ij_ is the phenotype of individual i on day t. An estimate was obtained for the asymptote, the time of inflection and the scaling factor. Asym, xmid and scal describe the overall mean curve for this population. As_i_ (ASYM), xm_i_ (XMID) and sc_i_ (SCAL) describe the deviations of the overall curve for each individual.

Asreml [4] was used to determine the heritability of these latent variables (ASYM, XMID and SCAL) using a model including the overall mean and random 'animal' and residual effects which were assumed to be normally distributed i.e.* u ~ N(0,Aσ_a_^2^)* and* e ~ N(0,Iσ_e_^2^).*

### QTL identification

QTL identification was done using a Bayesian Variable Selection Method (BVSM) [5]. The following model was fitted to each of the parameters of the growth curve separately:

param = μ + Σ_k_ σ_k_X _k_ ά_k_ + Zu + e

Where:

*param* is ASYM, XMID or SCAL for each individual and where terms* Σ_k_ σ_k_X _k_ ά_k_* fit marker association effects, where* ά_k_* is a vector with the allele substitution effects, with* ά_k_ ~N(0, I)* and* σ_k_* is a scaling factor that shrinks allele effects and models the variance explained by the marker. The scaling factors are conditionally estimated as simple Normally distributed regressions, and can be interpreted as a standard deviation (hence the symbol *σ*).* Zu* fits polygenic background effects with *u~N(0,Aσ_u_^2^)* with A the numerator relationship matrix between individuals derived from pedigree records. The error vector is* e~N(0,Iσ_e_^2^)*.

In the BVSM the shrinkage of allele effects, through the scaling factors* σ_k_*, is done in a dualistic manner by applying a mixture distribution on the scaling factors that heavily shrinks the effects for most of the markers, effectively removing most of the markers from the model. Only a small part of the marker effects are less severely shrunken, identifying the markers with important associations. This prior mixture distribution is a mixture of a Normal and a Truncated Normal distribution:

where the first distribution is referred to as the "null" distribution that models the majority of markers with (virtually) no effect using π_0_ =0.70 and setting* σ_g0_^2^* very small. Here* σ_g0_^2^* was set to 0.01*(variance of the trait)/( π_ 0_*(number of markers)), so that the markers in this group will jointly explain no more than 1% of variance in the trait. The second distribution models the markers with important effects. For this second distribution a truncated Normal is used so that the signs of the estimated allele effects will be identifiable, and the parameter* σ_g1_^2^* is estimated from the data, using a flat prior. In this case with relative few markers π_ 0_/ π_ 1_ was set at 0.7/0.3. For the mixture prior, the model estimates a "mixture indicator" which for each marker indicates whether it was estimated to belong to the first distribution or the second distribution. The first distribution is indicated by 0 and the second one with 1, so that, after averaging in the MCMC, a value ranging from 0 to 1 which is a posterior probability for each marker to have a large effect (i.e. the probability to belong to the second distribution) and can be used for model selection [5].

Using a simultaneous fit of all markers as in the BVSM can cause the signal of a QTL to be spread over multiple markers, i.e. several markers may get a moderate posterior probability for association, but none of the markers may have a very high posterior probability. Although this properly indicates the uncertainty about a QTL position, the evidence for presence of a QTL in the region may still be high, i.e. a *group* of markers (but no individual marker) may have a high joint posterior probability for association. In order to retrieve the evidence for association of groups of markers two approaches were used: (1) haplotypes in a group of markers were recoded into "alleles" of a pseudo marker and these new pseudo markers were analyzed; and (2) a post- marker-analysis (PMA) was performed on the patterns of mixture indicators generated in the Markov chain by grouping marker signals in windows. Both methods are capable of identifying haplotypes or marker windows which have a high probability of having a signal, although each underlying single marker may only show a moderate signal. In the second approach, the primary joint Gibbs samples for the mixture indicators were used, which take account of the switches for adjacent markers being on or off, to derive the joint probability for having a signal in a window. If the mixture indicators show that more than one SNP within a window (of maximal 9 SNPs) has a high probability of being in the model, this is counted to determine the probability of multiple QTLs.

### Applied MCMC techniques:

All samplers are single site Gibbs samplers. The particular parameterization with scaling factors was chosen so that scaling factors* σ_k_* can be sampled as "regressions" from Normal distributions and with Normal prior distributions. A speeded-up version for models with the mixture prior was implemented in which marker effects are only updated with 20% probability if the marker is "not associated" (comes from the first mixture). Markers which are "associated" are always updated, and markers neighbouring ("associated" markers are updated with probability* 0.8^d^* where* d* is the ordinal distance to the associated marker, up to* d=7,* after which the update probability falls back to the default 20%.

### Identification of associated markers

As indicated above, the posterior probability for a marker to come from the second mixture distribution can be used for model selection. We used two approaches to determine a cut-off on these posterior probabilities for the selection of significant associations, denoting the estimated posterior probability by  and the prior probabilities used in the model by* π_0_* and* π_1_.*

1. Analogous to the computation and use of the Bayes factor between two models we used here a "parameter-wise Bayes Factor" (pwBF) as the odds ration between posterior and prior probabilities for an individual marker:

Using guidelines by Kass and Raftery [6] to judge Bayes Factors, a value above 3.2 is "substantial", a value above 10 is "strong", and a value above 100 is "decisive".

2. Based on Conlon et al. [5] for a group of markers the sum of  probabilities can also be used to indicate the false discovery rate (FDR):  is the probability of erring when selecting a marker with posterior probability  as true. Hence when the top* m* markers are selected, the FDR in this group is .

## Results

In Table 1 the mean and phenotypic variance for the 3 latent variables: ASYM, XMID and SCAL (i.e. the parameters of the logistic growth curve) are given as well as their genetic parameters that were estimated using the standard animal model with ASreml. Heritabilities were around 48% for ASYM and SCAL while the heritability for XMID was approximately 24%. ASYM was genetically positively correlated with XMID and SCAL while the later two were not correlated. The genetic correlation could be due pleiotropic QTLs or to QTLs in close linkage disequilibrium. The assumption of a logistic growth curve underlying the data could be incorrect which could cause a spurious phenotypic correlation among the parameters but would less likely affect a genetic correlation.

**Table 1:** Mean, phenotypic variance and genetic parameters for the latent variables based on 5 observations per individual (n=1000). Heritabilities are on the diagonal and genetic correlations are below the diagonal (standard errors in brackets).

**latent variable**	**mean**	**Phenotypic**	**genetic parameters**		
		**variance**	**ASYM**	**XMID**	**SCAL**

asymptote (ASYM)	34.5	81.4	0.48 *(0.12)*		
inflection point (XMID)	415.4	126.7	0.42 *(0.21)*	0.24 *(0.08)*	
scaling factor (SCAL)	112.9	46.2	0.33 *(0.21)*	-0.02 *(0.25)*	0.48 *(0.12)*

Table 2 shows for each of the latent variables (ASYM, XMID and SCAL) the loci where markers picked up a significant amount of the variance indicating the presence of QTLs. Separate analyses were run for single SNPs as well as for haplotypes containing 2 SNPs.

**Table 2:** Loci associated with latent variables: asymptote (ASYM), inflection point (XMID) and scaling factor (SCAL) using haplotypes consisting of 1 and 2 SNPs.

**latent variable**	**SNPs/haplotype**	**Locus**	**pwBF**	**Prob(2ndMix)**	**FDR**

ASYM	1SNP	all_0.4447	231.0	1.00	0.00
		all_1.0359	231.0	1.00	0.00
		all_1.0516	231.0	1.00	0.00
		all_1.8834	103.7	0.98	0.01
		all_3.7168	24.2	0.91	0.02

	2 SNPs	all_0.4447+1	231.0	1.00	0.00
		all_1.0243+1	231.0	1.00	0.00
		all_1.8574+1	44.3	0.95	0.02
		all_1.0516+	9.3	0.80	0.06
		all_3.7168+1	1.3	0.36	0.18

XMID	1 SNP	all_0.4153	231.0	1.00	0.00

	2 SNPs	all_0.4029+1	23.6	0.91	0.09

SCAL	1SNP	all_1.4852	231.0	1.00	0.00
		all_0.9137	231.0	1.00	0.00
		all_3.0827	71.5	0.97	0.01
		all_3.0411	67.1	0.97	0.02
		all_3.8701	48.4	0.95	0.02
		all_2.3108	6.1	0.72	0.06
		all_4.3212	3.3	0.59	0.11
		all_3.9476	1.7	0.42	0.17
		all_4.3915	1.2	0.33	0.23

	2 SNPs	all_0.9137+1	231.0	1.00	0.00
		all_2.3108+1	21.0	0.90	0.05
		all_1.4829+1	18.9	0.89	0.07
		all_3.8701 + 1	12.2	0.84	0.09
		all_3.0480+1	3.8	0.62	0.15
		all_0.4447+1	3.8	0.62	0.19
		all_3.0813+1	2.9	0.55	0.23
		all_4.2975+1	2.5	0.52	0.26
		all_3.0128+1	1.5	0.39	0.30
		all_3.8360+1	1.1	0.33	0.33
		all_3.1467+1	1.0	0.31	0.37
		all_ 2.4607+1	1.0	0.31	0.39

**Figure 1:**
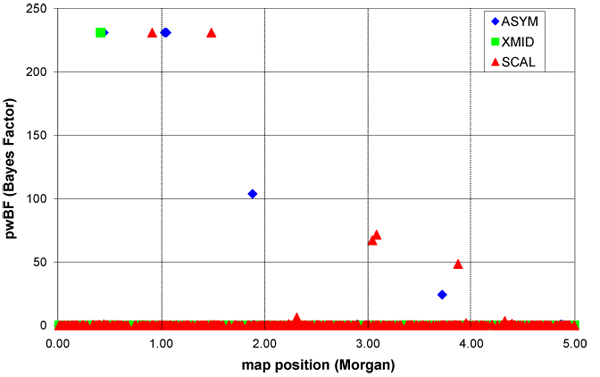
Bayes Factor (pwBF = posterior odds/ prior odds) for SNP association with each of the latent variables: ASYM, XMID and SCAL.

In Figure 1 a graphical overview is given of the position of the QTLs with a high Bayes factor (pwBF). Four QTLs affected ASYM and the two most prominent ones (equally important) were found on chromosome 1 and 2 (positions 0.04447 and 1.0359-1.0516) while a QTL, half as important, was found at the end of chromosome two (position 1.8834). A small QTL affecting ASYM was detected on chromosome 4 (position 3.7168). For XMID a single QTL was detected on chromosome one (position 0.04153). For SCAL four QTLs were detected. Two large ones on chromosome 1 and 2 (position 0.9137 and 1.4852 respectively). Two smaller QTL were detected on chromosome four (position 30411-30827 and 3.8701). The positions of QTL for ASYM and XMID on chromosome one were near. Co- segregation of alleles for the two QTL probably cause the genetic correlation observed. Co-segregation of QTL-alleles for ASYM and SCAL on chromosome two and four might explain the genetic correlation between the two parameters. Table 3 shows the region which were 'switched on' in a large number of the MCMC- samples. This analysis was based on analysis involving single SNPs. The results are very consistent with the previous analysis; however it indicates that the position of the QTL with a larger effect was more precise for ASYM and SCAL. The probability of more than one QTL in a region also increased when the size of the QTL was smaller. For XMID the position of the QTL was less clear according to the PMA- analysis and it also indicated a second QTL on the same chromosome. It could indicate that at least two QTL for XMID were segregating and due to co-segregation of positive/negative alleles for the two QTLs the positions remained obscure. Based on the results in Table 3 the two QTLs positions were between 0.3401 and 0.4831.

**Table 3:** Post-marker-analysis (PMA) results: region where a significant signal was observed for each of the three latent variables: asymptote (ASYM), inflection point (XMID) and scaling factor (SCAL). Region size indicates the number of SNPs in a window.

**latent variable**	**region size**	**Pr(1)^a^**	**Pr(>1)^b^**	**FDR**	**Marker Start**	**Marker End**

ASYM	1	1.00	0.00	0.00	all_0.4447	all_0.4447
	2	1.00	0.00	0.00	all_1.0359	all_1.0516
	5	1.00	0.18	0.00	all_1.8834	all_1.9011
	10	0.91	0.09	0.02	all_3.5612	all_3.7344

XMID	10	0.98	0.04	0.02	all_0.4029	all_0.4831
	7	0.98	0.03	0.02	all_0.3410	all_0.3970

SCAL	1	1.00	0.00	0.00	all_1.4852	all_1.4852
	1	1.00	0.00	0.00	all_3.0827	all_3.0827
	3	1.00	0.09	0.00	all_3.8517	all_3.8701
	1	1.00	0.00	0.00	all_0.9137	all_0.9137
	5	0.97	0.16	0.01	all_2.2809	all_2.3180
	5	0.75	0.23	0.05	All_4.1332	all_4.2895

^^a^^ probability of presence of a QTL

^^b^^ probability of more than one QTL

For ASYM the pwBF indicated two QTLs on position 1.0359 and 1.0516. From Table 3, however, it is clear that there is only one QTL in that region, i.e. the probability of more than one QTL is close to zero. The reverse is true for ASYM on position 1.8834 where the window-analysis showed a probability of 0.18 of having more than one QTL indicating that there might be 2 very close QTLs affecting ASYM. On chromosome 5 no QTL was detected for any of the latent variables.

## Discussion

The direct analysis of repeated measurements would not be a simple task because repeated observations will have strong correlations in time and typically show an increasing variance. An indirect approach as used here, which analyzes latent parameters of growth curves, is a much cleaner approach, obtaining variables that are less correlated, have constant variance, and possibly are also biologically more meaningful. For such an analysis of latent variables we used here a two step procedure, first defining latent variables and subsequent marker analyses of these variables, which is most likely not optimal. We envision that in the future they can be combined in a single analysis especially in a Bayesian context because it allows for hierarchical modeling as was shown by Yang and Xu [2]. In a single model the latent parameters can be modeled as well as the marker effect affecting these latent parameters. However, our software does not (yet) allow for this type of model. No test was applied to determine if QTL were pleiotropic or just in close LD. However, the software used to simulate the data set had not been used to generate such data sets (Coster, personal communication).

The Bayesian approach was found useful for multi-QTL mapping and obtains a good resolution, with suggestions for multiple closely linked QTLs. Simulation studies have also shown that the main advantage of this Bayesian approach is in the accuracy of QTL location (Sahana et al. in preparation). A multi-variate multi-QTL approach as applied by Meuwissen and Goddard [7] was not considered because this approach models variance-covariance matrices with multivariate Wishart distributions, which gives problematic convergence for the analysis of multiple highly correlated traits, such as the repeated weight measures in this study.

The Bayesian approach when run on single SNPs has the property that a QTL signal can be spread over multiple SNPs, thus masking possible important effects. This signal diffusion can be caused by uncertainty about the location of a QTL with a small effect, but also the signal of a 'large' QTL can get spread out over a group of strongly co-linear SNPs. The use of either haplotypes, or a post-marker-analysis that aggregates signals in windows appears a good approach to retrieve again clear QTL signals. The windowing approach has the advantage that only a single analysis needs to be made which can be post-analyzed with various window-settings. In these windows also the probability of having more than 1 signal can be computed which is lost when using haplotypes. The QTL for ASYM on position 1.8834 a loss of signal was observed, where the windowing approach indicated the possibility of 2 QTLs. Two closely positioned QTLs with effects in repulsion might explain this effect.

In Table 4, the size of the QTL and the true location, which were provided by the organizers after the workshop, are given in the first two columns. Six QTL were simulated for each parameter of the logistic growth curve which was used to simulate the phenotypes. The latter 5 columns contain the estimated location and their significance levels based on our analysis for each of the 18 QTL. In some QTL regions more than one SNP was identified in our analysis, these are also shown in Table 4. All QTL for ASYM and SCAL were actually found using the Bayesian algorithm, but for ASYM and SCAL two QTL did not meet our significance threshold of 0.05 for FDR. The five smaller QTL for XMID were not identified because there was virtually no information in the data to identify them as was shown during the workshop.

**Table 4:** Comparison of true and estimated location of all simulated QTL and their significance.

**latent**	**variance**	**true**	**Estimated**		**significance**		
**variable**	**of QTL**	**location**	**location**	**difference**	**pwBF**	**P(2ndMix)**	**FDR**

ASYM	0.586	0.4245	all_0.4447	0.0225*	231.000	1.000	0.000
	0.141	1.0455	all_1.0359	0.0096*	231.000	1.000	0.000
			all_1.0516	0.0061	231.000	1.000	0.000
	0.074	1.8864	all_1.8834	0.0030*	103.727	0.978	0.006
	0.066	3.6979	all_3.7168	0.0189*	24.182	0.912	0.022
	0.051	4.7719	all_4.8695	0.0976	0.737	0.240	0.145
	0.082	2.8984	all_2.8962	0.0022	0.721	0.236	0.233

XMID	0.644	0.5425	all_0.4153	0.1272*	231.000	0.994	0.006
			all_0.5309	0.0116	0.033	0.014	0.738
			all_0.5365	0.0060	0.019	0.008	0.823
			all_0.5381	0.0044	0.014	0.006	0.847
			all 0.6062	0.0637	0.009	0.004	0.880
			all_0.8210	0.2785	0.043	0.018	0.656
	0.064	3.3652	all_3.4010	0.0358	0.048	0.020	0.493
			all_3.3746	0.0094	0.019	0.008	0.789
	0.075	4.5971	all_4.7248	0.1277	0.014	0.006	0.866
	0.070	1.3302	all_1.5364	0.2062	0.009	0.004	0.892
	0.070	2.0686	all_2.1531	0.0845	0.009	0.004	0.952
	0.077	2.5609	all_2.5961	0.0352	0.009	0.004	0.960

SCAL	0.096	1.4889	all_1.4852	0.0037*	231.000	0.998	0.002
	0.467	0.8765	all_0.9137	0.0372*	231.000	1.000	0.001
	0.119	3.0962	all_3.0827	0.0135*	71.506	0.968	0.011
			all_3.0411	0.0551	67.111	0.966	0.017
	0.131	3.8639	all_3.8701	0.0062*	48.391	0.954	0.023
	0.094	2.2622	all_2.3108	0.0486	6.121	0.724	0.065
	0.092	4.3148	all_4.3212	0.0064	3.308	0.586	0.115

* indicates estimated significant QTL (FDR < 0.05)

## Conclusions

Repeated observations on individuals were affected by at least nine QTLs. For most QTL a precise location could be determined. The QTL for the inflection point (XMID) was difficult to pinpoint and might actually exist of two (or more) closely linked and segregating QTL on chromosome one.

## Competing interests

The authors declare that they have no competing interests.

## Authors' contributions

HH and LJ conceived the project. LJ developed the software (iBay). HH analyzed the data and together they wrote the paper.
